# ObsTIVA‐UK: a service evaluation of obstetric total intravenous anaesthesia in the United Kingdom

**DOI:** 10.1002/anr3.12293

**Published:** 2024-05-08

**Authors:** Y. Metodiev, H. A. Iliff, B. Sharif, S. F. Bell, C. Oliver, L. de Lloyd, ObsTIVA‐UK Collaborators Group

**Affiliations:** ^1^ Department of Anaesthesia University Hospital of Wales Cardiff UK; ^2^ School of Medicine Cardiff University Cardiff UK; ^3^ Department of Anaesthesia University Hospital of Wales Cardiff UK; ^4^ School of Medicine Cardiff University Cardiff UK

**Keywords:** anaesthesia, caesarean, intravenous, newborn, obstetric anaesthesia

## Abstract

We conducted a prospective observational service evaluation across the United Kingdom on the use of total intravenous anaesthesia (TIVA) for obstetric surgery between November 2022 and June 2023. The primary aim was to describe the incidence of TIVA for obstetric surgery within participating units, with secondary aims to describe maternal and neonatal postoperative recovery indicators. Of 184 maternity units in the United Kingdom, 30 (16%) contributed data to the service evaluation. There were 104 patients who underwent caesarean delivery under TIVA and 19 patients had TIVA for other reasons. Infusions of propofol and remifentanil were used in 100% and 84% of cases, respectively. Fifty‐nine out of 103 live neonates (57%) required some form of respiratory support. Of the neonates with recorded data, 73% and 17% had Apgar scores < 7 at 1 and 5 min respectively. No neonates were recorded to have Apgar scores < 7 at 10 min. Further prospective research is required to investigate the impact of obstetric TIVA on maternal and neonatal outcomes and inform best practice recommendations.

## Introduction

Advances in obstetric anaesthesia, in particular replacement of general anaesthesia (GA) with neuraxial anaesthesia as the default approach for caesarean delivery (CD), have contributed to major improvements in maternal and neonatal outcomes. Nevertheless, GA remains a vital technique, particularly in emergency situations [1, 2]. General anaesthesia typically utilises intravenous induction and volatile maintenance, but the use of total intravenous anaesthesia (TIVA) has rapidly increased over the past decade in the United Kingdom (from 8% to 26% of all GAs) [3]. The extent to which this translates to obstetric practice is unclear: case reports and single‐centre case series describing TIVA for obstetric anaesthesia have been published [4, 5, 6, 7, 8, 9, 10, 11, 12, 13, 14], but no systemwide evaluation has been conducted, and the impact of TIVA on maternal and neonatal outcomes remains unknown [15]. We designed a multicentre service evaluation to describe the incidence and practice of TIVA for obstetric surgery and immediate maternal and neonatal recovery outcomes, in participating centres in the United Kingdom.

## Methods

This prospective multicentre service evaluation was registered with Cardiff and Vale University Health Board (CAVUHB) (3650‐06/07/2022), and approved according to local procedures in participating centres. Data sharing agreements (compliant with information governance principles and General Data Protection Regulation) between CAVUHB and participating sites were completed. The Research and Development Department of CAVUHB deemed that ethical approval was not required.

All 184 NHS obstetric anaesthetic departments in England, Wales, Scotland and Northern Ireland were eligible to participate. Direct contact was made with 100 departments through trainee research networks, websites of local obstetric anaesthesia societies, and personal contacts. The project was promoted on the social media platform X (formally Twitter) with further information made available on the project website (https://www.obstiva.com). All eligible departments were encouraged to make enquiries if interested.

Data collection was via three inputs: an initial site registration survey completed by local project leads; anonymised case record forms; and a closing survey summarising the obstetric and anaesthetic activity in the local obstetric service during the data collection period. Patients who received TIVA for elective or emergency CD; or obstetric surgery within 24 h of birth; or were converted from regional anaesthetic to TIVA before/during surgery were included. Cases of non‐obstetric surgery during pregnancy or which involved conversion from inhalational anaesthesia to TIVA after delivery of the fetus were excluded. In addition to routinely recorded medical and obstetric characteristics, information was collected regarding indications for GA and reasons for choosing TIVA. Anonymised data were entered by local collaborators into a secure online REDCap® database (REDCap Consortium, Vanderbilt University, Nashville, Tennessee, USA).

The primary aim was to describe the incidence of TIVA for obstetric surgery within participating units, with secondary aims to describe maternal and neonatal postoperative recovery indicators and TIVA practice for obstetric surgery.

Non‐CD TIVA cases and cases involving neonates who were stillborn or born before initiating TIVA were excluded from analyses of maternal and neonatal outcomes. Data were analysed using Microsoft Excel (Microsoft, Inc., Redmond, USA) and JASP version 0.17.3 (University of Amsterdam, The Netherlands). Cohorts and incidence are reported using descriptive statistics.

## Results

**Figure 1:**
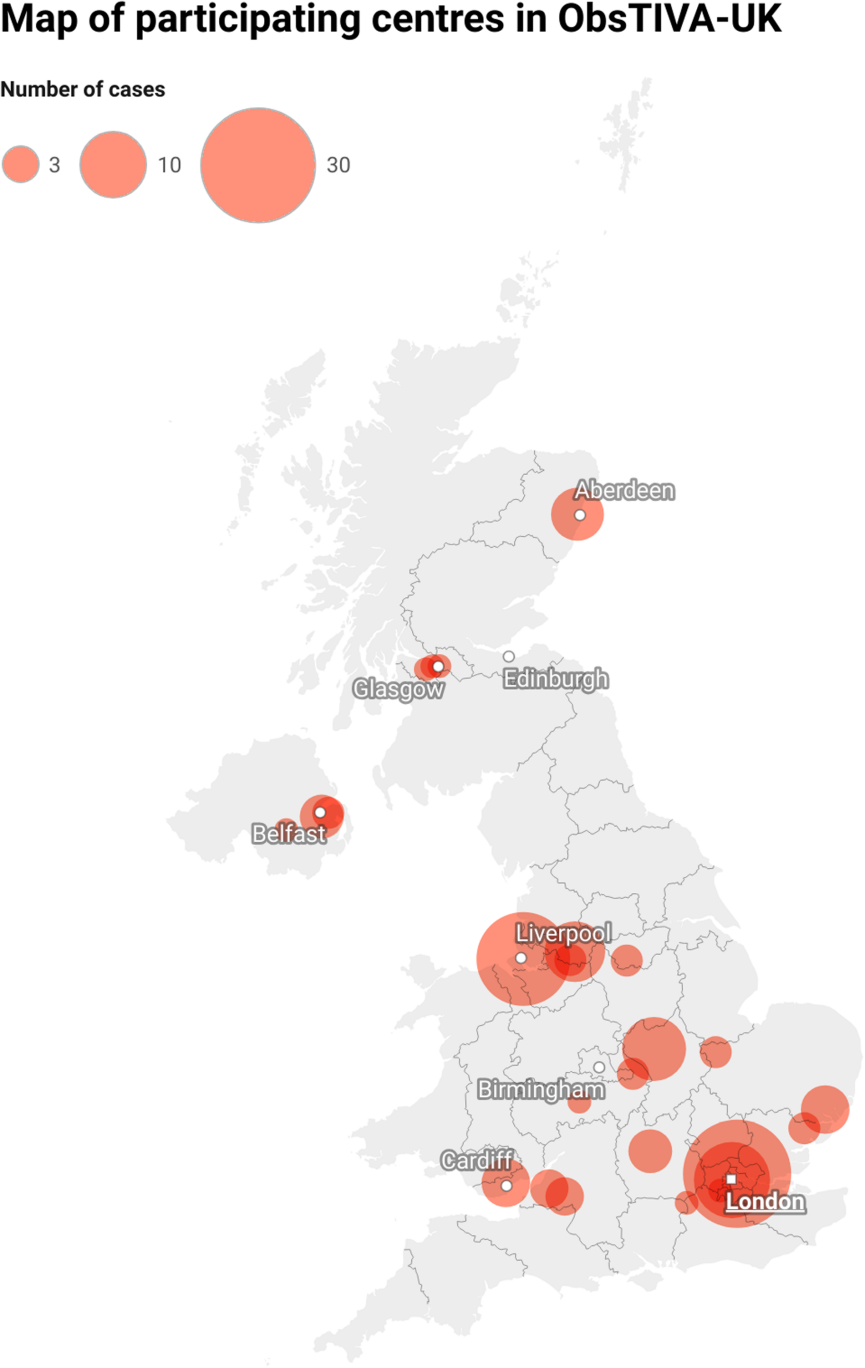
Map of participating centres in ObsTIVA‐UK. The size of each circle is proportional to the number of cases contributed to the project by each site.

Of 184 maternity units in the United Kingdom, 30 (16%) contributed data to the service evaluation between November 2022 and June 2023. The remaining departments declined, did not complete required agreements, or did not express interest in participating. Participating hospitals varied in size in terms of birth rate per annum: < 3000 (n = 2); 3000–5000 (n = 15); 5000–7000 (n = 10); > 7000 (n = 3). The geographical distribution of participating sites and the number of cases reported are shown in Figure 1.

During the period of the evaluation, 90,782 births were reported in participating units, with CD as the mode of birth for 37,281 (41%). There were 1877 GAs for obstetric surgical procedures (not limited to CDs), of which 123 utilised TIVA (6.6% of obstetric GAs). There were 104 patients who underwent CD under TIVA (0.3% of all CDs) which accounted for 85% of all recorded obstetric surgical cases in our dataset. Nineteen patients had TIVA for reasons other than CD: manual removal of placenta (n = 7); examination under anaesthesia (n = 6); perineal repair (n = 4); vaginal birth (n = 2).

Patient and obstetric characteristics, and indications for GA for CD and TIVA are shown in Table 1. Details of TIVA for CDs by classification of urgency are shown in Table 2.

**Table 1:** Patient characteristics, indications for general anaesthesia and total intravenous anaesthesia. Data are presented as mean (SD, [range]) or incidence (proportion, %).

	Category 1	Category 2	Category 3	Category 4	Total
	N = 9	N = 25	N = 21	N = 49	N = 104
Age, years	31 (4, [25–38])	34 (5, [23–43])	30 (5, [19–40])	34 (5, [23–42])	33 (5, [19–43])
Most recent weight, kg	75 (14, [55–100])	81 (13, [58–113])	78 (26, [54–180])	83 (32, [43–260])	81 (26, [43–260])
Most recent BMI, kg.m^−2^	29.6 (4.7, [21.5–35.7])	30 (4.9, [22.3–37.5])	29 (8.6, [21.1–63])	30.4 (10.4, [17.4–83.9])	30 (8.5, [17.4–83.9])
Gestation, weeks^+days^	38^+1^ (3^+1^, [31^+6^–41^+6^])	34^+5^ (4^+4^, [25^+0^–41^+3^])	35^+1^ (4^+1^, [26^+1^–39^+6^])	36^+6^ (1^+4^, [33^+1^–40^+1^])	36^+4^ (3^+3^, [25^+0^–41^+6^])
Singleton pregnancy	9 (100%)	25 (100%)	19 (90%)	47 (96%)	100 (96%)
Twin pregnancy	0	0	2 (10%)	2 (4%)	4 (4%)
PAS	0	2 (8%)	2 (10%)	7 (14%)	11 (11%)
Indication for GA					
Medical	2 (22%)	7 (28%)	5 (24%)	10 (20%)	24 (23%)
Urgency	6 (67%)	4 (16%)	0	0	10 (10%)
Patient choice	2 (22%)	4 (16%)	10 (48%)	24 (49%)	40 (39%)
Contraindication for RA	2 (22%)	13 (52%)	7 (33%)	14 (29%)	36 (35%)
Failed RA	0	3 (12%)	2 (10%)	4 (8%)	9 (9%)
Conversion from RA	0	3 (12%)	1 (5%)	1 (2%)	5 (5%)
Reason for TIVA					
Neurological disorder	0	3 (12%)	2 (10%)	8 (16%)	13 (13%)
Malignant hyperthermia	0	0	1 (5%)	0	1 (1%)
Cardiac disorder	0	3 (12%)	0	1 (2%)	4 (4%)
Anaesthetist preference	8 (89%)	22 (88%)	14 (67%)	43 (88%)	87 (84%)
Severe PONV	0	1 (4%)	0	0	1 (1%)
Risk of PPH	2 (22%)	9 (36%)	7 (33%)	17 (35%)	35 (34%)

BMI, body mass index; PAS, placenta accreta spectrum; GA, general anaesthesia; RA, regional anaesthesia; TIVA, total intravenous anaesthesia; PONV, postoperative nausea and vomiting; PPH, postpartum haemorrhage.

**Table 2:** Details of TIVA technique, drugs used, airway management. Results are presented as incidence (proportion, %) or mean (SD, [range])*.*

	Category 1	Category 2	Category 3	Category 4	Total
	N = 9	N = 25	N = 21	N = 49	N = 104
Propofol used	9 (100%)	25 (100%)	21 (100%)	49 (100%)	104 (100%)
Propofol infusion method					
TCI	9 (100%)	24 (96%)	21 (100%)	49 (100%)	103 (99%)
Simple infusion	0	1 (4%)	0	0	1 (1%)
Propofol TCI model					
Marsh	4 (44%)	14 (58%)	11 (52%)	35 (71%)	64 (62%)
Schnider	5 (56%)	9 (38%)	9 (43%)	13 (27%)	36 (35%)
Eleveld	0	1 (4%)	1 (5%)	1 (2%)	3 (3%)
Propofol maintenance dose, μg.ml^−1^	4 (0.7, [3–5])	4.2 (0.9, [2.8–6])	4.1 (1.2, [2.6–8])	4.3 (0.8, [2.5–6])	4.2 (0.9, [2.5–8])
Opioid used at induction*	8 (89%)	21 (84%)	17 (81%)	48 (98%)	94 (90%)
Remifentanil	6 (67%)	20 (95%)	17 (100%)	44 (92%)	87 (92%)
Alfentanil	1 (11%)	1 (5%)	0	2 (4%)	4 (4%)
Fentanyl	1 (11%)	0	0	2 (4%)	3 (3%)
Morphine	1 (11%)	0	0	0	1 (1%)
Remifentanil infusion method					
TCI	6 (100%)	20 (100%)	17 (100%)	43 (98%)	86 (99%)
Simple infusion	0	0	0	1 (2%)	1 (1%)
Remifentanil TCI model					
Minto	6 (100%)	20 (100%)	16 (94%)	43 (100%)	86 (99%)
Eleveld	0	0	1 (6%)	0	1 (1%)
Remifentanil maintenance dose, ng.ml^−1^	4.1 (0.7, [3–5])	4.6 (0.9, [3–6])	4.7 (1.3, [1.5–6.5])	4.7 (1.4, [2–8])	4.6 (1.3, [1.5–8])
Administration of induction drugs					
Pump bolus	0	13 (52%)	15 (71%)	34 (69%)	62 (60%)
Manual bolus	9 (100%)	10 (40%)	4 (19%)	9 (18%)	32 (30%)
Hybrid technique	0	2 (8%)	2 (10%)	6 (12%)	10 (10%)
Dedicated intravenous cannula	6 (67%)	20 (80%)	17 (81%)	26 (53%)	69 (66%)
Dedicated giving set	9 (100%)	22 (88%)	19 (90%)	46 (94%)	96 (92%)
Cannula visible	8 (89%)	24 (96%)	21 (100%)	45 (92%)	98 (94%)
pEEG monitoring	9 (100%)	23 (92%)	21 (100%)	49 (100%)	102 (98%)
BIS	4 (44%)	15 (60%)	13 (62%)	42 (86%)	74 (72%)
Narcotrend	5 (56%)	8 (32%)	5 (24%)	6 (12%)	24 (24%)
Other	0	0	3 (14%)	1 (2%)	4 (4%)
Antiemetics	9 (100%)	21 (84%)	21 (100%)	49 (100%)	100 (96%)
Ondansetron	9 (100%)	19 (90%)	20 (95%)	43 (88%)	91 (91%)
Dexamethasone	9 (100%)	19 (90%)	16 (76%)	36 (73%)	80 (80%)
Cyclizine	0	0	2 (10%)	1 (2%)	3 (3%)
Metoclopramide	0	1 (5%)	0	0	1 (1%)
Vasopressors	3 (33%)	15 (60%)	15 (71%)	33 (67%)	66 (67%)
Phenylephrine	2 (67%)	11 (73%)	13 (87%)	29 (88%)	55 (83%)
Norepinephrine infusion	0	2 (13%)	0	3 (9%)	5 (8%)
Metaraminol	1 (33%)	3 (20%)	2 (13%)	2 (6%)	8 (12%)
Ephedrine	0	1 (7%)	0	1 (3%)	2 (3%)

TCI, target‐controlled infusion; pEEG, processed electroencephalography; BIS, bispectral analysis.

* One patient received more than one opioid at induction of anaesthesia.

Anti‐emetic medication was given to 100/104 (96%) patients who underwent CD under TIVA. Data on postoperative nausea and vomiting (PONV) within the first six postoperative hours after CD was reported in 101/104 cases (97%): there was no PONV in 88/101 (88%); nausea in 10/101 (10%); and nausea and vomiting in 3/101 (3%). The median recorded blood loss (IQR, [range]) during CD was 600 ml (438–1000, [152–8000]).

There were 108 births under TIVA in this service evaluation. Neonates born before initiating TIVA (n = 2, 2%) or those who were stillborn (n = 3, 3%) were not included in the analysis of neonatal outcomes (Table 3). Fifty‐nine out of 103 neonates (57%) required some form of respiratory support, while 35/103 (34%) did not require respiratory support. Data were unavailable for 9/103 (9%). Most respiratory interventions were inflation breaths (42/59, 71%), followed by the application of continuous positive airway pressure (18/59, 31%). Eight neonates out of 103 (8%) required tracheal intubation, and 15 (15%) required intensive care admission which was not pre‐planned. Of the neonates requiring tracheal intubation, 7/8 (88%) were born prematurely (<37 weeks gestation), as were 8/15 (53%) of the neonates admitted to intensive care.

**Table 3:** Outcomes of neonates born under TIVA. Data are presented as median (IQR, [range]) or incidence/cases with data entry (proportion, %).

	Elective CD	Non‐elective CD	All CD
Apgar score at 1 min	n = 45	n = 43	n = 88
	6 (3–7, [0–9])	6 (4–7, [1–10])	6 (4–7, [0–10])
Apgar score at 5 min	n = 45	n = 42	n = 87
	8 (7–9, [4–10])	8 (7–9, [3–10])	8 (7–9, [3–10])
Apgar score at 10 min	n = 36	n = 36	n = 72
	10 (9–10, [7–10])	10 (8–10, [7–10])	10 (9–10, [7–10])
Apgar score < 7 at 1 min	33 (73%)	31 (72%)	64 (73%)
Apgar score < 7 at 5 min	9 (20%)	6 (14%)	15 (17%)
Apgar score < 7 at 10 min	0	0	0

CD, caesarean delivery; TIVA, total intravenous anaesthesia.

## Discussion

This multicentre cohort report demonstrates that TIVA accounted for 6.6% of obstetric GAs in participating units in the United Kingdom. Total intravenous anaesthesia was administered for all categories of CD, using varied techniques, with the majority administered for elective CD. Data were provided by 30 (16%) obstetric anaesthetic units in the UK making this the largest evaluation of TIVA use in obstetric anaesthesia to date.

A limitation of this report is that units are self‐selected and therefore data may be biased towards respondents who hold strong opinions on TIVA, and thus our data may not be representative of anaesthetic practice in all UK obstetric units. Results may also be skewed towards a small number of practitioners within these sites since ‘anaesthetist's preference’ was a reason for TIVA in 82% of cases (Table 1). In addition, data collection was limited to routine data within 6 h of GA emergence and was not designed to describe outcomes such as accidental awareness under GA or patient‐reported outcomes.

The strength of this report is that it is the largest of its kind to date. It shows that TIVA was used for 6.6% of obstetric GAs in participating units, with the majority used for non‐time‐critical surgery. Interest in obstetric TIVA is increasing [16], and use in non‐obstetric surgery is becoming commonplace, with training in the technique now a requirement in the UK anaesthetic training curriculum. If the use of TIVA increases to the extent that it becomes the majority technique for GA, anaesthetists may have less confidence and experience with volatile anaesthesia, and individual preference for this technique may mean that it becomes the default in obstetric practice.

When compared to regional anaesthetic techniques, GA is associated with worse short‐term neonatal outcomes such as Apgar scores < 7 at 1 min and increased need for respiratory support [17, 18, 19, 20, 21]. In this study (Table 3), the incidence of Apgar scores < 7 at 1 min was higher than previously described, occurring in 73% of neonates born during TIVA, half of whom were born by elective CD. Apgar scores improved at 5 and 10 min. Unexpected admissions to neonatal intensive care occurred in 15% of cases. Although these data are observational and information on the duration of exposure to TIVA was not collected, the high requirement for intervention in neonates born under TIVA is concerning and requires further investigation.

Odor et al reported on GA in obstetrics during a national study, conducted in England between 2017 and 2018; they found that 15% of GAs were for elective (category 4) CD, and 51% were for emergency/category 1 CD [22]. In our evaluation (Table 1), approximately half of the cases were in women with no clinical urgency (category 4), and maternal preference was the leading indication for GA (39%); three times the incidence reported by Odor et al [22]. In 34% of cases, PPH was recorded as a reason for using TIVA; whilst there is a theoretical advantage of using TIVA to avoid the uterine relaxant effects of volatile anaesthesia, no large studies have investigated this in clinical practice [23].

As far as we are aware, there are currently no established guidelines or protocols available to guide TIVA use in obstetric practice, and it is therefore unsurprising that the implementation of TIVA in this report was variable. Of note, some anaesthetists reported a ‘hybrid’ method for increasing the speed of drug delivery in urgent cases, involving the administration of a manual bolus decanted from the pump (e.g. via a three‐way tap) during the induction phase of the target‐controlled infusion (TCI). Setting up a TCI is safety‐critical, and can be time‐consuming, which may have precluded its use in the most urgent clinical scenarios [24].

The use of processed electroencephalography (pEEG) monitoring during TIVA with neuromuscular blocking agents is recommended [25] and 102/104 (98%) patients in our evaluation had pEEG monitoring placed during TIVA (Table 2). However, the physiological changes of pregnancy and obstetric pathology such as preeclampsia may influence EEG activity and interpretation, which has not been well characterised in the literature [26]. Nevertheless, TIVA has the theoretical advantage of reducing the incidence of accidental awareness under GA by avoiding the ‘gap’ between intravenous induction and the onset of volatile anaesthesia [27], although whether this advantage would be delivered in practice is unknown.

In this study there was a high incidence of Apgar scores < 7 and a need for respiratory support in neonates born under TIVA. If TIVA is chosen for CD, this should be communicated to the attending neonatal clinician to ensure that an appropriately skilled team are immediately available. Techniques in TIVA may require modification to optimise outcomes in obstetric practice and this is a priority for future study. A prospective study is required to investigate the impact of obstetric TIVA on maternal and neonatal outcomes and inform best practice recommendations.

## Supporting information


**Appendix S1.** List of collaborators.

## References

[1] Bamber JH , Lucas DN , Plaat F , Russell R . Obstetric anaesthetic practice in the UK: a descriptive analysis of the National Obstetric Anaesthetic Database 2009–14. British Journal of Anaesthesia 2020; 125: 580–587. 32736825 10.1016/j.bja.2020.06.053

[2] Juang J , Gabriel RA , Dutton RP , Palanisamy A , Urman RD . Choice of anesthesia for cesarean delivery: an analysis of the national anesthesia clinical outcomes registry. Anesthesia and Analgesia 2017; 124: 1914–1917. 28098588 10.1213/ANE.0000000000001677

[3] Kane AD , Soar J , Armstrong RA , et al. Patient characteristics, anaesthetic workload and techniques in the UK: an analysis from the 7th National Audit Project (NAP7) activity survey. Anaesthesia 2023; 78: 701–711. 36857758 10.1111/anae.15989

[4] Javed U , Bhatia K . Total intravenous anaesthesia for caesarean delivery: incidence, maternal and neonatal outcomes from a tertiary unit. International Journal of Obstetric Anesthesia 2023; 57: 103933. 37866971 10.1016/j.ijoa.2023.103933

[5] Van De Velde M , Teunkens A , Kuypers M , Dewinter T , Vandermeersch E . General anaesthesia with target controlled infusion of propofol for planned caesarean section: maternal and neonatal effects of a remifentanil‐based technique. International Journal of Obstetric Anesthesia 2004; 13: 153–158. 15321393 10.1016/j.ijoa.2004.01.005

[6] Llopis JE , Garcia‐Aguado R , Sifre C , et al. Total intravenous anaesthesia for caesarean section in a patient with Marfan's syndrome. International Journal of Obstetric Anesthesia 1997; 6: 59–62. 15321314 10.1016/s0959-289x(97)80055-6

[7] Xiao W , Zhao L , Wang F , Sun H , Wang T , Zhao G . Total intravenous anesthesia without muscle relaxant in a parturient with amyotrophic lateral sclerosis undergoing cesarean section: a case report. Journal of Clinical Anesthesia 2017; 36: 107–109. 28183545 10.1016/j.jclinane.2016.10.009

[8] McCarroll CP , Paxton LD , Elliott P , Wilson DB . Use of remifentanil in a patient with peripartum cardiomyopathy requiring caesarean section. British Journal of Anaesthesia 2001; 86: 135–138. 11575392 10.1093/bja/86.1.135

[9] Mertens E , Saldien V , Coppejans H , Bettens K , Vercauteren M . Target controlled infusion of remifentanil and propofol for cesarean section in a patient with multivalvular disease and severe pulmonary hypertension. Acta Anaesthesiologica Belgica 2001; 52: 207–209. 11534314

[10] Fujita M , Satsumae T , Tanaka M . General anesthesia using remifentanil for cesarean section in a parturient with Marfan syndrome associated with heart failure due to severe mitral regurgitation. Masui. The Japanese Journal of Anesthesiology 2016; 65: 530–534. 27319100

[11] Macfarlane AJ , Moise S , Smith D . Caesarean section using total intravenous anaesthesia in a patient with Ebstein's anomaly complicated by supraventricular tachycardia. International Journal of Obstetric Anesthesia 2007; 16: 155–159. 17270419 10.1016/j.ijoa.2006.07.009

[12] Niki A , Ochiai D , Iwai M , Sato Y , Yoshino K , Yamada T . Management of pregnancy complicated by central core disease. International Journal of Obstetric Anesthesia 2020; 43: 25–26. 32570046 10.1016/j.ijoa.2020.05.004

[13] Foster RN , Boothroyd KP . Caesarean section in a complicated case of central core disease. Anaesthesia 2008; 63: 544–547. 18412656 10.1111/j.1365-2044.2007.05411.x

[14] Zuccolotto EB , Pagnussatt Neto E , Nogueira GC , Nociti JR . Anesthesia in pregnant women with HELLP syndrome: case report. Brazilian Journal of Anesthesiology 2016; 66: 657–660. 10.1016/j.bjane.2014.05.013 27793243

[15] Metodiev Y , Lucas DN . The role of total intravenous anaesthesia for caesarean delivery. International Journal of Obstetric Anesthesia 2022; 51: 103548. 35490115 10.1016/j.ijoa.2022.103548

[16] Scale R , Johnson‐Hughes H , Metodiev Y . Availability of total intravenous anaesthesia for obstetric surgery: a survey of UK practice. European Journal of Anaesthesiology 2024; 41: 146–148. 37158658 10.1097/EJA.0000000000001855

[17] Gwanzura C , Gavi S , Mangiza M , et al. Effect of anesthesia administration method on Apgar scores of infants born to women undergoing elective cesarean section. BMC Anesthesiology 2023; 23: 142. 37106343 10.1186/s12871-023-02098-w PMC10134612

[18] Sung TY , Jee YS , You HJ , Cho CK . Comparison of the effect of general and spinal anesthesia for elective cesarean section on maternal and fetal outcomes: a retrospective cohort study. Anesthesia and Pain Medicine 2021; 16: 49–55. 33389986 10.17085/apm.20072 PMC7861904

[19] Beckmann M , Calderbank S . Mode of anaesthetic for category 1 caesarean sections and neonatal outcomes. Australian and New Zealand Journal of Obstetrics and Gynaecology 2012; 52: 316–320. 22676478 10.1111/j.1479-828X.2012.01457.x

[20] Kim WH , Hur M , Park SK , et al. Comparison between general, spinal, epidural, and combined spinal‐epidural anesthesia for cesarean delivery: a network meta‐analysis. International Journal of Obstetric Anesthesia 2019; 37: 5–15. 30415797 10.1016/j.ijoa.2018.09.012

[21] Ring L , Landau R , Delgado C . The current role of general anesthesia for cesarean delivery. Current Anesthesiology Reports 2021; 11: 18–27. 33642943 10.1007/s40140-021-00437-6 PMC7902754

[22] Odor PM , Bampoe S , Moonesinghe SR , et al. General anaesthetic and airway management practice for obstetric surgery in England: a prospective, multicentre observational study. Anaesthesia 2021; 76: 460–471. 32959372 10.1111/anae.15250

[23] Thind AS , Turner RJ . In vitro effects of propofol on gravid human myometrium. Anaesthesia and Intensive Care 2008; 36: 802–806. 19115648 10.1177/0310057X0803600609

[24] Al‐Rifai Z , Mulvey D . Principles of total intravenous anaesthesia: practical aspects of using total intravenous anaesthesia. BJA Education 2016; 16: 276–280.

[25] Nimmo AF , Absalom AR , Bagshaw O , et al. Guidelines for the safe practice of total intravenous anaesthesia (TIVA): joint guidelines from the Association of Anaesthetists and the Society for Intravenous Anaesthesia. Anaesthesia 2019; 74: 211–224. 30378102 10.1111/anae.14428

[26] Corner H , Barley M , Metodiev Y . The use of processed electroencephalography (pEEG) in obstetric anaesthesia: a narrative review. International Journal of Obstetric Anesthesia 2023; 54: 103650. 36934515 10.1016/j.ijoa.2023.103650

[27] Palmer JHM , Pandit JJ . AAGA during induction of anaesthesia and transfer into theatre. In: Pandit JJ , Cook TM , eds. NAP5: accidental awareness during general anaesthesia in the United Kingdom and Ireland. London: Royal College of Anaesthetists and Association of Anaesthetists of Great Britain and Ireland, 2014: 63–76.

