# Supplementary material for: ObsTIVA‐UK: a service evaluation of obstetric total intravenous anaesthesia in the United Kingdom
Source: Anaesth Rep. 2024 May 8;12(1):e12293. doi: 10.1002/anr3.12293 (PMC11078484; doi:10.1002/anr3.12293)
Supplement: Supplementary file 1 — Appendix S1. List of collaborators. [file ANR3-12-e12293-s001.pdf]

ObsTIVA-UK Collaborators List:

Welsh Anaesthesia Audit Research and Engagement Network (WAAREN)

Pan-London Perioperative Audit and Research Network (PLAN)

West of Scotland Anaesthetic Trainees Research Network (WoSTRAQ)

Severn Trainee Anaesthetic Research Group (STAR)

Adam Green

Adam McConville

Adetokunbo Owolabi

Aiswarya Kunnumpurath

Alexander Sharp

Alison Blair

Amarjeet Patil

Amrutha Vishwanathan

Andrew Clark

Andrew Ling

Ania Dean

Anya Sheltawy

Azka Afzal

Banu Ergezer

Ben Gupta

Charles Philpott

Charlotte Green

Chris Marsh

Chris McGrath

Chung Chow

Cliff Shelton

Daisy Riddle

Damien Hughes

Daniel Brimfield

Daniel Bruynseels

Daniel Watkins

Danielle Eusuf

David Golding

David Green

David Quinn

Desire Onwochei

Devan Williams

Dominic Allen

Edward Gill

Ee-Min Wong

Emily Robson

Fleur Roberts

Georgia Halladay

Grainne Garvey

Helen McNamara

Iain Mactier

Inthekab Ali Mohamed Ali

Jack Tooze

Jan Man Wong

Jason Scott

Jasprit Sidhu

Jonathan Holmes

Julia Blackburn

Julia Niewiarowski

Kailash Bhatia

Karan Verma

Karen Sykes

Kate Bosworth

Kaushik Makam

Kieran Hardern

Lauren Barraclough

Malcolm Broom

Mark Entwistle

Matthew Sinnott

Megan Griffiths

Min Ji Lee

Natalie Hills

Natalie Silvey

Nilesh Sonawane

Nilofer Shaik

Oliver Williams

Paul Maguire

Paul Sharpe

Paul Wyatt

Peter Eskander

Philip Jackson

Prerna Mehrotra

Priya Shinde

Radha Pagedar

Ramesh Vedagiri Sai

Rebecca Summers

Reshma Patel

Richard George

Richard Morrison

Robert Wilkinson

Rose smith

Roy Williamson

Samuel Naylor

Sarah McDonald

Sarah Todhunter

Sebastian Murray

Serena Bourke

Shashikant Yegnaram

Tabitha Tanqueray

Thomas Gilkes

Thomas Moorhen

Tom Pettigrew

Victoria Randall

Vijeta Mahinthan

Yize Wan
